# Design of a Shape-Memory-Alloy-Based Carangiform Robotic Fishtail with Improved Forward Thrust

**DOI:** 10.3390/s24020544

**Published:** 2024-01-15

**Authors:** Mithilesh Kumar Koiri, Vineet Dubey, Anuj Kumar Sharma, Daniel Chuchala

**Affiliations:** 1 Nims Institute of Engineering and Technology, Nims University, Jaipur 303121, India; koiri2013@gmail.com; 2 School of Mechatronics Engineering, Symbiosis Skills and Professional University, Pune 412101, India; 3 Centre for Advanced Studies, Dr. A. P. J. Abdul Kalam Technical University, Lucknow 226031, India; sharmaanuj79@gmail.com; 4 Institute of Manufacturing and Materials Technology, Faculty of Mechanical Engineering and Ship Technology, Gdańsk University of Technology, 1/12 G. Narutowicza Street, 80-233 Gdańsk, Poland

**Keywords:** robotic fish, SMA, underwater robot, origami, smart material

## Abstract

Shape memory alloys (SMAs) have become the most common choice for the development of mini- and micro-type soft bio-inspired robots due to their high power-to-weight ratio, ability to be installed and operated in limited space, silent and vibration-free operation, biocompatibility, and corrosion resistance properties. Moreover, SMA spring-type actuators are used for developing different continuum robots, exhibiting high degrees of freedom and flexibility. Spring- or any elastic-material-based antagonistic or biasing force is mostly preferred among all other biasing techniques to generate periodic oscillation of SMA actuator-based robotic body parts. In this model-based study, SMA-based spring-type actuators were used to develop a carangiform-type robotic fishtail. Fin size optimization for the maximization of forward thrust was performed for the developed system by varying different parameters, such as caudal fin size, current through actuators, pulse-width modulation signal (PWM), and operating depth. A caudal fin with a mixed fin pattern between the Lunate and Fork “Lunafork” and a fin area of approximately 5000 mm^2^ was found to be the most effective for the developed system. The maximum forward thrust developed by this fin was recorded as 40 gmf at an operation depth of 12.5 cm in a body of still water.

## 1. Introduction

Currently, several underwater robots are being developed for exploring the behaviour and continuous observation of the health conditions of smaller aquatic animals [1]. These robots are also used for discovering new aquatic species, exploring oil, minerals, and other discoveries that would be helpful for the welfare of humanity. A critical review of most of the robotic fish developed in the last decade showed that servo motor-based robotic fish have mostly been used for developing underwater robotic fish. Sound, vibration, unbalancing during operation, and complexity in body structural design are some common limitations of motor-based robotic fish. The silent, smooth, simple, and effective operation of smart material actuators such as shape memory alloys (SMAs) [2,3,4,5,6], dielectric elastomer actuators (DEA) [7,8], ionic polymer–metal composite (IPMC) actuators [9,10], etc., has attracted the attention of researchers for developing smart-material-based robotic fish [11]. It has also been observed that among all smart-actuator-based robotic fishtails, SMA spring-actuator-based robotic fishtails develop high forward thrust [12]. At the same time, they have a higher power-to-weight ratio and take up minimal operating space [13]. Furthermore, corrosion is the biggest challenge to be addressed while developing an underwater robotic fish. The SMA actuator showed high biocompatibility and excellent corrosion resistance [14]. Among all other SMAs, Nitinol is chosen, as it has excellent electrical and mechanical properties, a long fatigue life, high corrosion resistance, and is readily available on the market [15]. However, less forward thrust and corresponding speed and a small operating cycle of fins are some limitations in the cases of smart-actuator-based robotic fish.

Shaw and Thakur [16] fabricated and studied a stretched SMA-wired actuator-based caudal fin for robotic fish. An SMA wire-based Bimorph mechanism (a cantilever used for actuation or sensing, having two active layers with a passive layer between two active layers for storing energy) was used to produce oscillation. The model (230 gm) achieved a maximum speed of up to 2.6 cm·s^−1^. Zhang et al. [17] used SMA plat strips for developing different robotic fish (3208 gm). The model achieved a maximum speed of 0.026 m·s^−1^. Manta ray robotic fish were developed by Wang et al. [18]. Here, stretched SMA wire (0.15 mm) was used to develop a flapping mechanism. A flexible robotic fish was developed, in which several wired actuators were attached along a flexible backbone made of ABS material. Each wire of the developed model produced a pull force of 321 gm [19]. Continuous caudal fin oscillation using SMA wire (0.1 mm) and a GE strip-based rocking–rocking mechanism was developed by Le et al. [2]. The model developed a maximum forward thrust of 0.004 N. Rossi et al. [20] developed gear- and motor-less robotic fish, in which stretched SMA wire is attached along a bending backbone made of a continuous deformable structure. Phamduy et al. [21] developed a carangiform type of robotic fish, having a body weight of 1.2 kg and a body length of 46 cm. Servo motors were used to produce undulation at the fishtail. The maximum speed achieved was noted to be 13.7 cm/s. Zhong et al. [22] developed wire-driven robotic fish; the measured weight and body length of these robotic fish were 0.5 gm and 310 mm, respectively. Two wires were used to undulate the fishtail using a servo motor. The maximum speed was 2.15 BL/s, and the flapping frequency was 3 Hz. Katzschmann [23] designed and developed a soft robotic fish using a hydraulic actuator. Piston-type hydraulic actuators were used to produce flapping of the fishtail. The body length of the robotic fish was 470 mm and the maximum speed achieved was 210 mm/s. Berlinger et al. [24] developed a DEA-based caudal fin attached at the rear end of the fish body. The weight of the robot was 115 gm, and the length was 100 mm. The maximum speed achieved was 55 mm/s. Le et al. [25] developed an SMA string-based robotic fish. The elastic properties of SMA wire were used to produce oscillation using a four-bar mechanism. The body size of the robotic fish was 26 cm, and the maximum speed achieved by the robot was 1.6 cm/s. Furthermore, Muralidharan and Palani [12] developed a sub-carangiform robotic fish using SMA spring-type actuators, having an actuator wire diameter of 0.77 mm and a coil diameter of 5.69 mm. The forward speed developed by the robotic fish was observed to be 24.5 mm·s^−1^. Coil-type SMA actuators were used in the robotic fish, and the developed forward thrust was 0.39 N. This was the largest value compared with the range of robotic fish propelled using SMA-based actuators. Despite the larger forward thrust, the forward speed was found to be very small due to the lower caudal fin oscillation frequency. At the same time, a large amount of energy dissipated due to direct contact of the SMA spring actuators with water.

In this study, our model was successfully realized and tested for forward thrust development by exploring varying sizes of caudal fins and depths of water. It focused on the optimization of SMA spring-based fishtail shapes and sizes for a given robotic fish. Maximum forward thrust was observed for a given fin size, heating time, cooling time, and water depth. An origami-based prismatic frustum-type skin was used to cover the SMA spring, as well as to produce an antagonistic force against the actuators. This reduces power loss due to the direct contact of SMA actuators with water. Mathematical modelling was developed, which demonstrated the methodology used to increase SMA-based fishtail oscillation. The technique of measuring the cyclic frequency of a SMA spring-based robotic system is also discussed. Initially, only four actuators (two on the right side and two on the left sides) were designed, and the central steel spring was replaced with an origami-based prismatic flexible skin. The study-based design and characterization of Nitinol SMA spring actuators have been performed [3]. After assembling all developed components, various actuators and sensors were connected to the controller. Here, the optimization variables involved the oscillation frequency, the area of the caudal fin, the operating power required, and the working depth. The objective of this study was to improve the flexibility of the fishtail, reduce power loss through the SMA spring actuators, and optimize the size of the caudal fin for maximum forward thrust developed by the given robotic system.

## 2. Materials and Methods

### 2.1. Actuator Design

**Figure 1:**
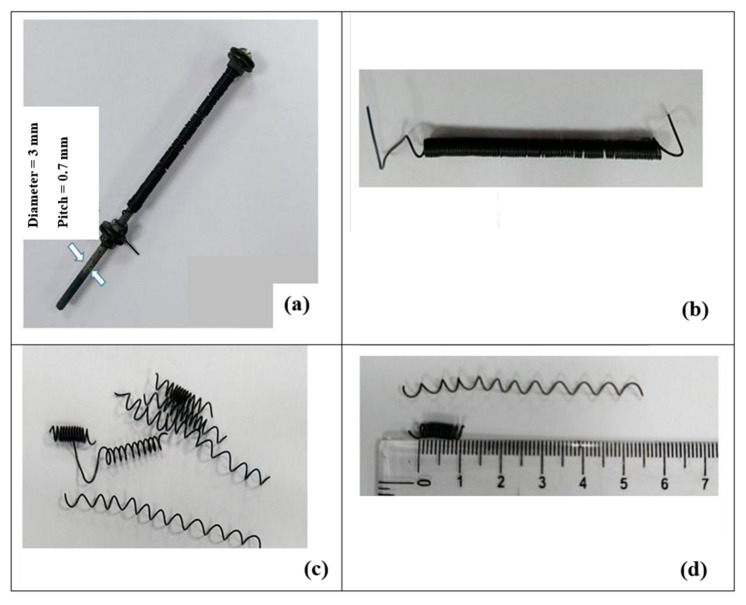
(**a**) SMA wire tightly wound around a bolt. (**b**) Trained SMA wire spring. (**c**) Trained SMA wire cut into pieces to form the SMA actuator. (**d**) Actuation capability of the designed SMA actuator.

**Figure 2:**
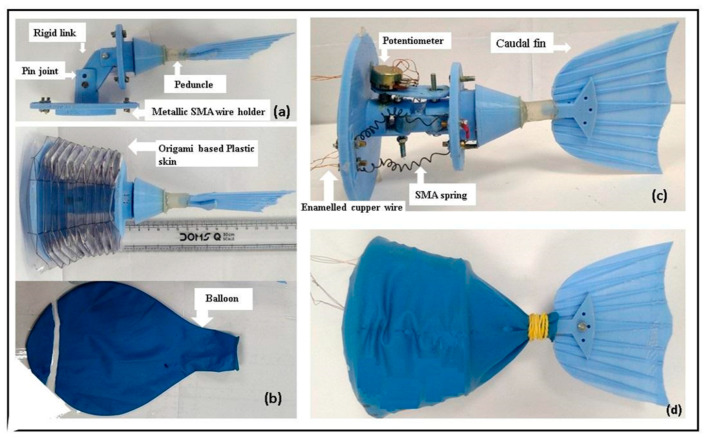
(**a**) The basic parts of the carangiform robotic tail body and the skeleton of the robotic tail. (**b**) The origami-based flexible skin made of plastic and a balloon for waterproofing the whole system. (**c**) The assembled inner part of the robotic fish containing an SMA spring, potentiometer, pin joint, and enamelled copper wire. (**d**) The finally developed 1/3 backward part of the robotic fish ready to test.

Firstly, SMA wires were procured, and their characteristics as an SMA spring-type actuator were studied. In order to achieve optimal actuation force, several experiments were performed on different SMA wires having varying diameters and lengths [3]. Among all SMA wires, Nitinol SMA wire with a wire diameter of 0.5 mm was selected for designing SMA actuators [26]. For developing the SMA spring-type actuator, wires were wound tightly around a bolt with a diameter of 3 mm (Figure 1a). Subsequently, the tightly held set of SMA wire was kept in the furnace and heated at a temperature of 450 °C for one hour, then cooled slowly in open air (Figure 1b). Then, based on the pre-design setup, the initial trained SMA spring was cut into pieces (Figure 1c). Finally, a trained SMA spring actuator was ready for further use. (Figure 2d).

Different tests pertaining to force, displacement, and power dissipation with respect to current and corresponding temperature were performed on the final SMA actuator. The characteristics and behaviour of the final actuator were used in the model. Specifications of the spring-type actuator are detailed in Table 1.

### 2.2. Tail Body and Caudal Fin Design

The size of the robotic fish was selected based on a review of previously developed similar kinds of robotic fish. It was observed that for a given forward thrust and corresponding speed, the body size of almost all the robotic fish lay in the range of 30 cm to 40 cm. It was decided to develop a carangiform-type robotic fish [27], with a body length of 39 cm and a flexible tail length of 13 cm. A streamlined body shape with a minimum (0.04) drag coefficient is selected. The model was designed on Solidworks (2018) and printed using a Makerbot 3D printer(Stratasys, Edina, MN, USA), using Poly Lactic acid (PLA) [28] as the work material. In order to cover the SMA actuator, an origami-based prismatic frustum and bellow-type skin, equivalent to a vertebral biasing spring for a carangiform type robotic tail, was developed (Figure 2b). This creates antagonistic force against the application of the unidirectional bending force produced by the SMA actuators. At the same time, it yields radial strength against the hydrostatic pressure developed during the operation. Temperatures of 80–90 °C are developed in each SMA actuator. Metallic wire holders are framed up to the cross-sectional body structure, as shown in Figure 2a. The oscillating part of the tail body is connected via a pin joint with the other part. The entire tail body is covered, in order to make the whole system waterproof. The rear part of the body structure accommodated the caudal fin attached to a flexible peduncle, which oscillated about the friction-less hinged joint to produce forward thrust. 

The caudal fin was attached to the body structure via a cylindrical peduncle made of Ethylene-vinyl acetate (EVA) copolymers. The caudal fin with a mixed fin pattern between the Fork and Lunate [29] was made of PLA. Table 2 shows the specifications of the robotic tail.

### 2.3. Experimental Setup Design

**Figure 3:**
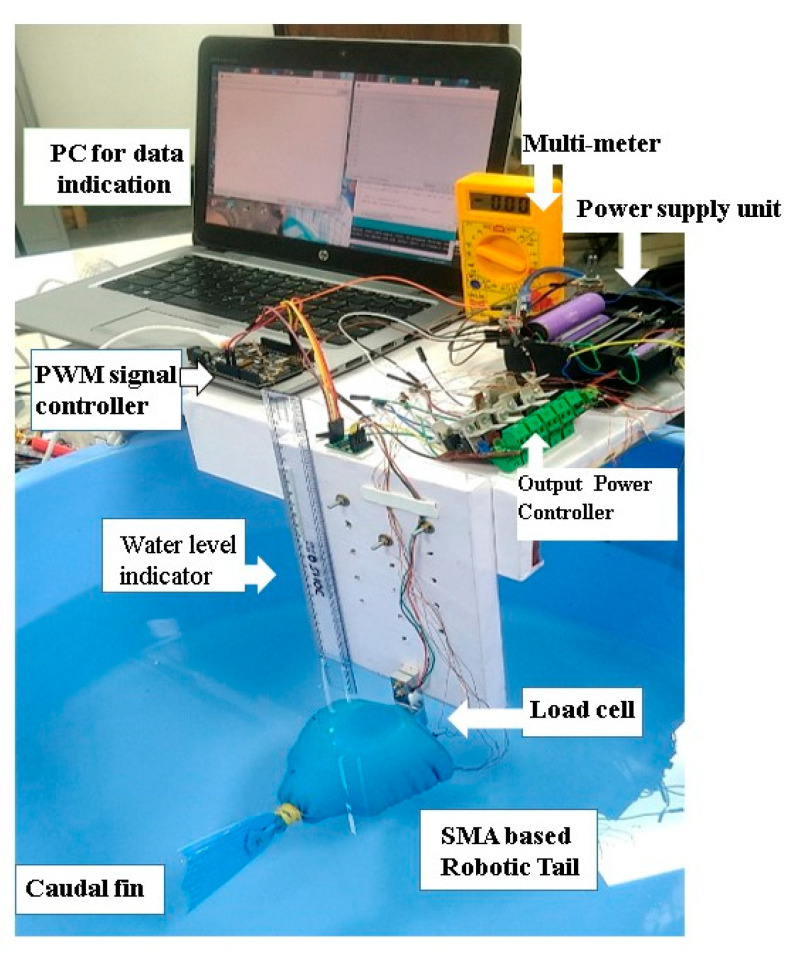
Experimental setup for measuring forward thrust developed by the SMA spring actuator-based robotic tail.

The experimental setup involved assembling of all the required devices and components, as shown in Figure 3. A DC power supply of 16V was used. The maximum output current was measured using a multi-meter. Initially, all the components were temporarily arranged on a wooden frame. A load cell (Xcluma 1 kg load cell weight sensor with HX711 ADC, Bytesware Electronics, Bengaluru, India) was used for measuring forward thrust. The depth of the tail was controlled manually. The turning angle of the fish tail was measured using a potentiometer attached inside the tail body; the corresponding frequency was measured using a stopwatch.

### 2.4. Mathematical Modelling and Characterization

**Figure 4:**
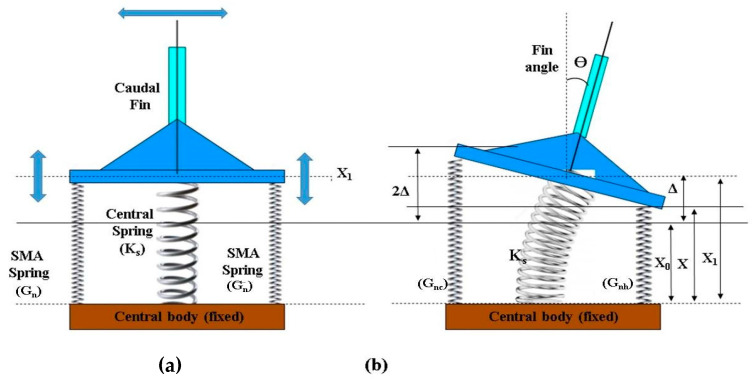
(**a**) The tested nonlinear behaviour of the 0.5 mm SMA (Nitinol) spring actuator and related terminologies. (**b**) Equivalent schematic diagram and simplified mathematical model of the robotic tail system.

To simplify motion analysis of the developed robotic fishtail (Figure 2d), a basic schematic diagram of the robotic fishtail system was drawn (Figure 4a,b). Caudal fin oscillation was produced by the linear alternating actuation of SMA spring actuators. The authors focused on the relationship between the motion of the fishtail, the shear modulus of the SMA spring, and the stiffness of the central spring (the bellow-type flexible origami skin behaved like a central spring).

In Figure 4, $x_{0}$ is the initial length of the pre-stretched SMA spring actuator, $x_{1}$ is the length of stretched SMA springs when they are fixed to the fishtail system, and the shear modulus of the stretched SMA spring is *G_n_*. The robotic tail system (Figure 4a) remained at its initial position ($x_{1}$) when no actuation of the actuators took place.

When SMA actuators on the right-hand side of the fishtail system were actuated, the caudal fin reached a new position (*x*). For this position, the forces developed by different springs can be determined.

The generalized equation of force developed by the hot spring (right-hand side) can be represented by

(1) $$F_{h}(x)=G_{nh}(x-x_{0})$$

At the same time, the equation of antagonistic force developed by the cold spring and central spring at the same position can be represented by

(2) $$F_{c}\left(x\right)=G_{nc}\left(x_{0}+2∆-x\right)+K_{s}\theta$$

where $G_{nh}$ and $G_{nc}$ are the shear modulus of the hot SMA spring and cold SMA spring, respectively, θ is the angle of deflection of the central spring (caudal fin), and $K_{s}$ is the stiffness of the central spring.

However, for the equilibrium of the fishtail system at this position, e.g., at $x_{2}$ (rightmost position of caudal fin),

(3) $$F_{h}\left(x_{2}\right)=F_{c}\left(x_{2}\right)\;$$

By solving the above equation, we can derive

(4) $$x_{2}=x_{0}+\frac{K_{s}\theta_{m}+2∆G_{nc}\;}{G_{nc}+G_{nh}}$$

Similarly, for position $x_{3}$ (leftmost position of caudal fin),

(5) $$x_{3}=x_{0}+\frac{K_{s}\theta_{m}+2∆G_{nh}\;}{G_{nc}+G_{nh}}$$

It is possible to determine out any positions between $x_{2}$ and $x_{3}$ using Equation (5). The maximum deformation from the mean position can be represented by

(6) $$\delta_{max}=\left(x_{3}-x_{0}\right)=\frac{K_{s}\theta_{m}+2∆K_{nh}\;}{G_{nc}+G_{nh}}$$

An oscillation stroke of the caudal fin is given by

(7) $$S=\left(x_{3}-x_{2}\right)=\frac{2∆{(G}_{nh}-G_{nc})\;}{G_{nc}+G_{nh}}\;$$

It is apparent from the robotic fishtail system that the deflection of origami below flexible skin (∆) is inversely proportional to the stiffness of the central spring ($K_{s}$):

(8) $$∆\;\propto \;\frac{1}{K_{s}}$$

By inserting the value of Equation (8) into Equation (7), the equation of oscillating stroke becomes

(9) $$S=\frac{2{(G}_{nh}-G_{nc})\;}{K_{s}(G_{nc}+G_{nh})}$$

Therefore, from the above results, it is clear that the oscillating stroke of the fin is inversely proportional to the stiffness of the central spring (bellow-type flexible skin) and the sum of the share modulus of hot and cold SMA springs. At the same time, the oscillating stroke is directly proportional to the difference in the shear modulus of hot and cold SMA spring actuators.

#### 2.4.1. Technique to Enhance the Cyclic Frequency

The mathematical model for the robotic fishtail is shown in Equation (9).

$$S\propto \frac{2{(G}_{nh}-G_{nc})\;}{K_{s}(G_{nc}+G_{nh})}$$

The oscillating frequency of caudal fin is the rate of cyclic change of stroke. From the above equation, it is clear that the rate of change in oscillating stroke is directly proportional to the difference of rate of change of rigidity of SMA spring actuators.

(10) $$\dot{S}\;\propto (\;\dot{G_{nh}}-\dot{G_{nc}})$$

It is observed that the rate of change in the rigidity of SMA spring actuators is directly proportional to the rate of heating of SMA wire actuators, and inversely proportional to the rate of cooling of SMA wire actuators:

(11) $$\dot{G_{n}}\;\propto \;\dot{Q_{h}}$$

(12) $$\dot{G_{n}}\;\propto \;\frac{1}{\dot{Q_{c}}}\;$$

However, it is very well established that heat dissipation by SMA wire is

(13) $$\dot{Q}=I^{2}R-hA\;(T_{SMA}-T_{amb})$$

where $I^{2}R$ is the heat power developed by DC current passing through the wire per unit time, h is the heat transfer coefficient, A is the surface area of the SMA wire actuator, $T_{SMA}$ is the temperature of SMA wire, and $T_{amb}$ is the temperature of the surrounding air [30].

During the heat dissipation by SMA wire, when it is lubricated by liquid mist, heat remaining or stored in the wire per unit time can be calculated as

(14) $$\dot{Q}=I^{2}R-mc_{p}\frac{dT}{dt}$$

where *m* is the mass of lubricated liquid and $c_{p}$ is the heat capacity of liquids.

For enhancing the rate of change in oscillating stroke or the oscillating frequency of SMA spring-actuator-based fishtail systems, the cooling rate need to be enhanced. Additionally, if the cooling rate increases, less heat will be stored in the SMA wire; therefore, damping of the caudal fin oscillation will not take place. Cooling of the SMA spring-type actuators can be enhanced by sprays of water mist, alcohol mist, or mist of any other liquid. The heat capacity ($c_{p}$) of the spraying materials should be high, but the lubricated boiling point should be lower.

#### 2.4.2. Forward Thrust Developed Due to Drag Force

The rowing speed of the caudal fin attached to the SMA spring actuators-based tail system is very slow. The forward thrust developed by the robotic fish is due to the drag force developed in the water. To develop forward thrust, SMA spring-type actuators apply alternative forces on each side of the fishtail. The tail structure is similar to a seesaw mechanism (Figure 4a). An electro-mechanical force is applied on either side, during the alternate actuation of SMA spring actuators. Considering the second-class lever mechanism, with force applied at the side ends, a fulcrum at the caudal fin, and load at the centre along the vertebrae of fish, the following equation can be written:

(15) $$\mathrm{The}\;\mathrm{mechanical}\;\mathrm{advantage}\;\mathrm{of}\;\mathrm{tail}\;\mathrm{structure}=\frac{forward\;thrust\;}{applied\;force\;by\;actuators}=\frac{r_{c}+r_{w}}{r_{c}}$$

(16) $$\mathrm{It}\;\mathrm{is}\;\mathrm{well}\;\mathrm{established}\;\mathrm{that}\;drag\;force\left(F_{d}\right)=\frac{1}{2}\;C_{d\;}\rho \;a_{fin}V^{2\;}$$

The applied force required by the actuators is equal to the drag force developed at the caudal fin to induce constant forward velocity of the robotic fish.

Thus, the forward thrust experienced by the robotic fish will be

(17) $$F_{t}=\left(F_{d}\right)\;\frac{r_{c}+r_{w}}{r_{c}}=\frac{r_{c}+r_{w}}{2r_{c}}\;C_{d\;}\rho \;a_{fin}V^{2\;}$$

where $a_{fin}$ is the area of the caudal fin and V is the rowing velocity of the caudal fin.

(18) $$\mathrm{Forward}\;\mathrm{thrust}\;\mathrm{experienced}\;\mathrm{by}\;\mathrm{the}\;\mathrm{robotic}\;\mathrm{fish}=\int \frac{r_{c}+r_{w}}{2r_{c}}\;C_{d\;}\rho a_{fin}V^{2\;}sin\;{2\theta }_{m}\;d\theta$$

From the Equation (18), it is clear that the forward thrust depends upon the size of the fishtail structure of robotic fishtail, drag coefficient, area of the fin, velocity of the fin, and the effective angle rowed by the fin, which also depends upon the flexibility of the fin.

Utilising Equation (18), the area ($a_{fin}$) of the fin can also be calculated if the other parameters are known.

## 3. Results

### 3.1. Calculation of Cyclic Frequency for a Given Signal

**Figure 5:**
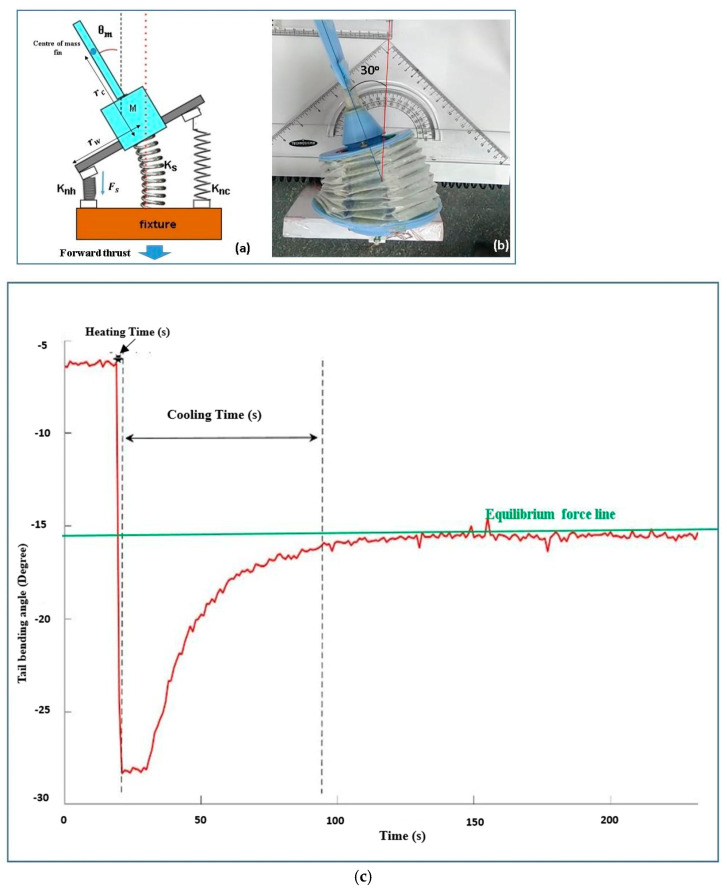
(**a**) Schematic of the robotic tail. (**b**) Working model of the robotic tail. (**c**) The signal obtained by the potentiometer attached inside the robotic tail using MATLAB.

An alternating square wave was generated with different cyclic frequencies. Figure 5c shows the characteristic graph plot (obtained using MATLAB R2018b) of an SMA spring attached to the left side of the fishtail system as an actuator. In this figure, the positive x-axis indicates the time, the positive y-axis indicates the turning angle in the right side, and the negative y-axis indicates the turning angle obtained on the left side. The turning angle was measured using a potentiometer as a radial position sensor. When a direct current passes through single or multiple SMA actuators, attached to any one side of the robotic body for duration of one or two seconds, a sudden contraction of the corresponding robotic tail takes place, because of the heat developed into that actuator.

Figure 5c depicts the signal when a 7.05-ampere current passes through the two parallelly connected SMA spring-type actuators for 2 s; sudden contraction of the actuators takes place due to heat developed in the wires. Furthermore, the very next moment, when the power supply is stopped, gradual cooling of the actuators takes place, and the corresponding angle of the caudal fin starts decreasing due to the antagonistic force developed by the central spring system. At a certain angle (represented by the green line, about 15 degrees) of the caudal fin, the force developed by the central spring and the force developed by the SMA spring actuators become equal to each other. This angle is denoted by a straight line called the equilibrium force line. The position of the equilibrium force line may change based on the number of SMA spring actuators used. The area below this line is called the dead zone, as no biasing force acts on the caudal fin until the application of the next actuation signal. The signal on the other side can also be analysed.

**Figure 6:**
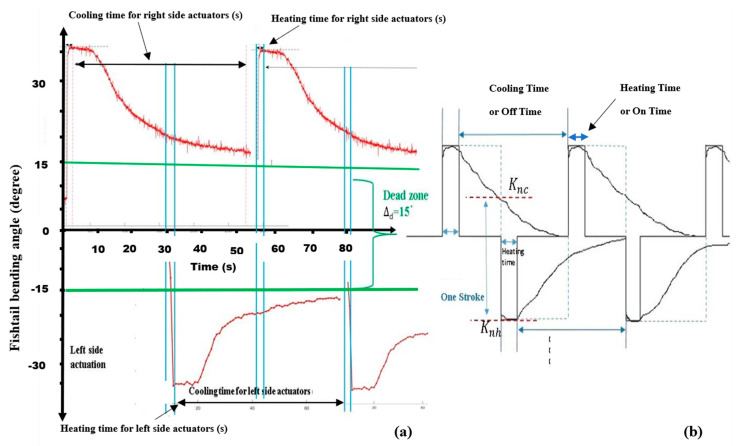
(**a**) The arrangement of actual signals obtained. (**b**) An ideal square wave generated to activate the SMA spring-actuator-based robotic tail.

As discussed earlier, the developed system requires an alternating square-wave-type signal for the continuous oscillation of the caudal fin. Based on the signals obtained for different actuators, the corresponding alternating square wave is mathematically calculated so that none of the actuators contain residual stress due to heat left in the actuator body before the next heating operation takes place, as shown in Figure 6a,b.

### 3.2. Fin Design and Test to Achieve Maximum Forward Thrust

As stated in the Introduction, a similar type of robotic fishtail was developed by Muralidharan and Palani [12], which produced a forward thrust of 39 gmf. In this study, the authors aimed to design a modified version of the carangiform-type robotic fish tail, which uses less operating power and can produce forward thrust of up to 40 gmf.

Based on the above information of the robotic system, the area of the fin may be calculated for improved forward thrust. For solely developing forward thrust ($F_{t}=40\;gmf$), the theoretical fin area ($a_{fin}$) can be calculated.

The maximum bending angle (Figure 5b) is observed: ${2\theta }_{m}=60^{{}^{\circ}}$.

Thus, the calculated area of the SMA spring-based fin ($a_{fin}$) will be

$$a_{fin}=\frac{\;F_{t}\;}{\frac{r_{c}+r_{w}}{2r_{c}}\;C_{d\;}\rho V^{2\;}\int \sin{2\theta }_{m}\;d\;\theta \;}$$

$$a_{fin}=\frac{0.4}{\frac{r_{c}+r_{w}}{2r_{c}}\;C_{d\;}\rho V^{2\;}\int \sin{2\theta }_{m}\;d\;\theta \;}$$

The linear velocity of the caudal fin can be calculated using the maximum swept angle, $\theta =\frac{\pi }{180}*60{}^{\circ}$; the angular velocity, $\omega =\frac{\theta }{t}$ rad/s, swept time (t) = 0.3 s (observed); and linear velocity, $V=r_{c}\omega \;m/s$ = 0.21 m/s.

Inputting the values of, $r_{c}=0.06\;m$, $r_{w}=0.03\;m$, $C_{d\;}=1.1(rectangular\;thin\;disk)$, $\rho =1000\;gk/m^{3}$, the area of the fin was calculated: 0.00637 m^2^ = 6370 mm^2^.

Based on the above theoretical results, a caudal fin with a mixed fin pattern between the Lunate and Fork “Lunafork” [29] was designed. The areas of the caudal fins are taken as fin a = 7000 mm^2^, fin b = 6000 mm^2^, fin c = 5000 mm^2^, and fin d = 4000 mm^2^.

**Figure 7:**
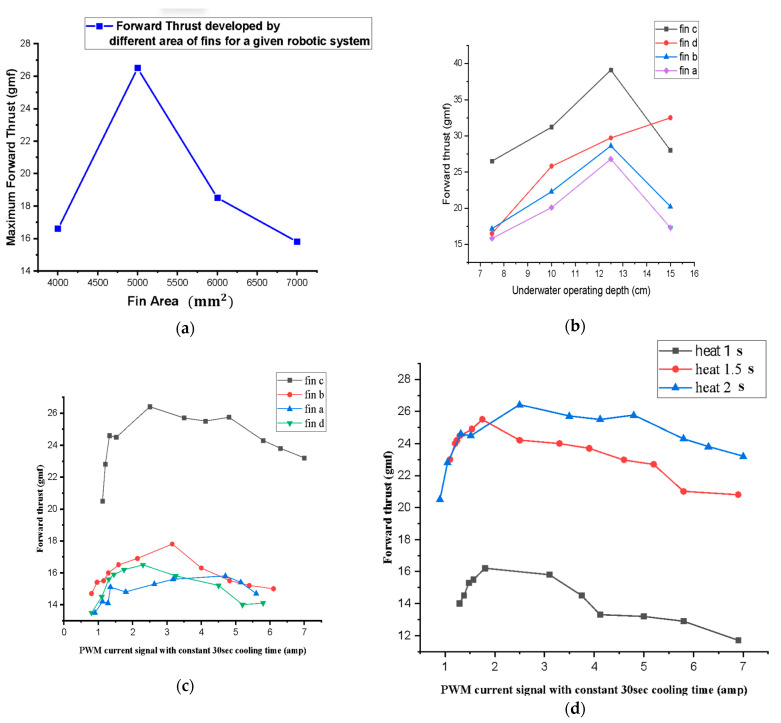
(**a**) Variation in forwarding thrust with the area of caudal fins. (**b**) Variation in forward thrust with depth. (**c**) Variation in forward thrust with the current for various caudal fin for a given robotic system. (**d**) Variation in forward thrust with the current for different heating times for a given robotic system (constant cooling time 30 s).

Different experiments were performed using the developed carangiform-type SMA-based robotic tail in various conditions; the corresponding results were plotted. Four similar types of caudal fins with different surface areas were designed in Solidworks and printed using a 3D printer. Figure 7a shows the variation in maximum forward thrust with the variation in the area of caudal fins for a given tail system and corresponding power supply.

For the given tail system receiving similar signals (same heating and cooling times), at comparable operating depths and environments, the 5000 mm^2^ caudal fin developed the highest forward thrust. The fins with an area less 5000 mm^2^ did not cause sufficient drag force, and hence, produced forward thrust. However, caudal fins with a surface area over 5000 mm^2^ did not receive sufficient power to overcome the drag force developed during operation.

For different caudal fins, the forward thrust is measured with the manual variation in depth of the robotic tail from the surface of the water (Figure 7b). The recorded values of forwarding thrust with operating depth are plotted. With the increment in depth, the forward thrust increased up to a certain value and then decreased. It is a well-known fact that hydrostatic pressure can be calculated as follows:

$$P=\rho \;g\;h_{d},\;\mathrm{where}\;h_{d}=operating\;depth$$

Thus, with depth, the hydrostatic pressure increases, which increases the corresponding forward thrust up to certain level due to the increased reaction force. However, over a certain depth, pressure is too high for the tail system to overcome. In this system, the maximum forward thrust was recorded up to 13 cm depth for fin c. The obtained results justify the fact that a given robotic fish system can only operate effectively up to certain depth.

Figure 7c shows the variation in forward thrust with current for different caudal fin areas in similar environmental conditions. Initially, when a very small current passed through the SMA actuators (two SMA spring-type actuators parallelly connected in our case), less heat develops in the wires, which results in less corresponding thrust. Additionally, the transition temperature of the SMA spring actuator is around 45–55 °C, which can be achieved by passing currents of 1 amp to 1.5 amp through each actuator.

After passing a certain amount of current through SMA actuators for 2 s (30 s was chosen for cooling the actuators in the air so that no stress remained in the actuators), the forward thrust started increasing. Increasing PWM direct current, from around 2 amperes to 3.5 amperes, the forward thrust increased. At the same time, it was found that increasing the heat at a constant cooling time reduced the forward thrust. With the increase in the area of the fins, initially, the forward thrust increased up to a certain value, but after that, it started reducing for the developed robotic system.

Figure 7d shows the variation in forward thrust with increasing heating time for the caudal fin. Although the PWM signal with 2 s heating and 30 s cooling developed the highest forward thrust up to a certain current range, above a certain current range, the forward thrust started reducing due to the insufficient cooling time.

Here, the whole current supplied at one time was divided into two parts for each SMA actuator. A maximum current of 7 ampere was supplied by the power system at one time, giving only 3.5 ampere current to each actuator, which took sufficiently longer to heat the actuators. This is the reason behind the increments in caudal fin rowing times and reductions in the expected forward thrust.

## 4. Conclusions and Future Scope

In this study, the SMA spring-actuator-based carangiform robotic tail design, corresponding mathematical modelling of the fishtail, square wave signal generation, drag-force-based caudal fin design, and their testing for better forward thrust have been performed. Based on the above design, modelling, experiments, and corresponding findings, the following conclusions can be drawn.

A fin area of approximately 6370 mm^2^ was calculated to provide a forward thrust of 0.4 N. For the developed robotic fishtail system, the optimal value of area was recorded to be around 5000 mm^2^. For the developed robotic system, a 12.5 cm depth was found to be most effective for the fish to move at maximum speed. At this depth, about 40 gmf of 0.4 N forward thrust was developed by fin c.

The low operating frequency of the SMA actuator-based system was found to be the biggest drawback. Based on the above techniques, fin shapes and sizes could be designed and developed for any specific robotic system. In turn, the oscillation frequency can be increased by optimizing the rate of rigidity change using various cooling methods. This technique can also be used in other SMA-based bioinspired robots inspired by different animals, such as starfish, jellyfish, and octopuses.

## Figures and Tables

**Table 1:** Specification of SMA spring actuator.

Name of Different Parameters	Specifications of SMA Actuators
SMA wire diameter	0.5 mm
Mean diameter of actuators	3.5 mm
Number of effective coils	10
The contracted length of the actuator	10 mm
Normal length	50 mm
Extension of actuator	60 mm
Total length of wire	110 mm

**Table 2:** Specification of the SMA-based robotic tail.

Name of Different Parts	Specifications of the Parts of Robotic Fish
Weight of the flexible tail	170 gm
Length	140 mm
Material used	PLA (tail body), EVA (peduncle), and PP (origami skin)
The maximum length of the axis of an elliptical cross-sectional plate	90 mm × 80 mm

## Data Availability

Data are contained within the article.
